# Supplementary material for: Protein arginine methyltransferase 5 (PRMT5) is an actionable therapeutic target in CDK4/6 inhibitor-resistant ER+/RB-deficient breast cancer
Source: Res Sq. 2023 Jul 10:rs.3.rs-2966905. Preprint. [Version 1] doi: 10.21203/rs.3.rs-2966905/v1 (PMC10371097; doi:10.21203/rs.3.rs-2966905/v1)
Supplement: 1 [file NIHPPRS2966905V1-supplement-1.pdf]

Supplementary Figure 1. *RB1* knockout in ER+ breast cancer cells confers resistance to CDK4/6 inhibitors

A) Immunoblot analysis of MCF-7 and T47D *RB1* wild-type (WT) and *RB1* knockout (RBKO) cell lysates. The lysates were probed with the indicated antibodies. **B-C)** Dose response curves to CDK4/6i. WT and RBKO cells of MCF-7 **(B)** and T47D **(C)** were treated with a dose range of abemaciclib, palbociclib, or ribociclib for 6 days. Cell viability was measured by the CyQuant assay (mean ± SD, n = 3). **D-E)** IC_{50} of abemaciclib, palbociclib, and ribociclib in WT and RBKO cells of MCF-7 **(D)** and T47D **(E)**. Data represent IC_{50} in μM and fold change in the parenthesis. ND: not determined.

Supplementary Figure 2. Sensitivity of ER+/*RB1* knockout (RBKO) breast cancer cells to PRMT5 inhibitor

A-B) Dose response curves of JNJ64619178 and **C-D**) GSK591. MCF-7 **(A)** and T47D **(B)** RBKO cells were treated with a concentration range of JNJ64619178 for 6 days. CAMA1 **(C)** and KPL1 **(D)** RBKO cells were treated with a concentration range of GSK591 for 6 days. Cell viability was measured by the CyQuant assay. Data represent mean ± SD (n = 3).

Supplementary Figure 3. Top 10 of upregulated Hallmark gene signatures upon *RB1* knockout.

A-B) Gene set enrichment analysis (GSEA) using RBKO vs WT RNA-seq data from MCF-7 **(A)** and T47D **(B)** cells. NES: normalized enrichment score; FDR: false discovery rate.

Supplementary Figure 4. *PRMT5* knockdown in *RB1*-deficient cancer cells results in growth inhibition and blocks G1- to S-phase cell cycle progression.

A) Immunoblot analysis of *RB1*-mutant lung cancer (H596, H1048 and H1155), prostate cancer (Du-145) and triple-negative breast cancer (MDA-MB-436) cells. Lysates were collected 3 days after transfection of control

siRNA (siCtrl) or a siRNA targeting *PRMT5* (siPRMT5) and then probed with antibodies as indicated. **B)** Growth of siCtrl- or siPRMT5-transfected *RB1*-mutant cancer cells. Cell number was counted using a Coulter counterafter 4-6 days after siRNA transfection. Data represent mean ± SD; ** (P <.01), *** (P <.001), **** (P <.0001), Student's t test. **C)** Cell cycle analysis. Cells were fixed 3-4 days after transfection of siCtrl or siPRMT5. Cells were stained with propidium iodide (PI) and then analyzed by flow cytometry Data represent mean ± SD (n = 3).

Supplementary Figure 5. ER+ breast cancer cells are sensitive to anti-estrogens irrespective of *RB1* status.

A) Monolayer growth of MCF-7 and T47D WT and RBKO cells treated with vehicle control (Veh), 10 nM fulvestrant (Fulv), E2-deprived IMEM (E2-) and 1 nM 17β-estradiol in E2-deprived IMEM (E2+). Cells were counted on day-7 using a Coulter counter. Data represent mean ± SD (n = 3); *** (P <.001), **** (P <.0001), one-way ANOVA with a Dunnett's post-hoc test. **B)** Estrogen responsive element (ERE) luciferase reporter assay. Cells were co-transfected with pGLB-MERE and internal control pCMV-Renilla. Next day, MCF-7 and T47D WT and RBKO cells were treated as indicated for 24 hours and then were subjected to dual-luciferase reporter assay. Data represent mean ± SD (n = 5); * (P <.05), ** (P <.01), *** (P <.001), **** (P <.0001), one-way ANOVA with a Dunnett's post-hoc test. **C)** Cell cycle analysis of MCF-7 and T47D WT and RBKO cells. Cells were treated as indicated for 3 days, fixed, and then stained with propidium (PI). Cell cycle analysis was conducted using flow cytometry (mean ± SD, n = 3).

Supplementary Figure 6. Characterization of the ER+/*RB1*-deleted PDX.

A) Immunoblot analysis of PDX lysates, probed with the indicated antibodies. **B)** IHC of total Rb on PDX FFPE sections. Pos: positive control.

Supplementary Figure 7. IHC of ER+/*RB1*-deficient tumors

A-B) Representative IHC images of SDMA, Erα, and Ki67 on FFPR sections of MCF-7_RBKO xenografts **(A)** and ER+/*RB1*-deleted PDXs **(B)**.
